# Case Report: Hyperthyroidism in a patient with spotty skin pigmentation and atrial myxoma (Carney complex): coincidence, association or cause?

**DOI:** 10.3389/fcvm.2026.1769687

**Published:** 2026-03-30

**Authors:** Donghua Pan, Shanshan Ding, Shitao Fang, Yulin Zhang

**Affiliations:** 1 The First College of Clinical Medical Science, China Three Gorges University, Yichang City, Hubei Province, China; 2 Department of Thoracic and Cardiovascular Surgery, Yichang Central People's Hospital, Yichang City, Hubei Province, China; 3 Department of Obstetrics and Gynecology, Yichang Central People's Hospital, Yichang City, Hubei Province, China

**Keywords:** atrial myxoma, cardiac surgical procedures, Carney complex, case, Graves’ disease

## Abstract

**Background:**

Carney complex (CNC) is a rare autosomal-dominant multiple neoplasia syndrome characterised by spotty skin pigmentation, cardiac myxomas and endocrine overactivity. Although thyroid follicular adenomas are common in CNC, the presence of CNC in combination with Graves' disease-related hyperthyroidism is rare, and the pathogenic association between the two conditions remains poorly understood.

**Case presentation:**

An 18-year-old Chinese woman presented with palpitations and worsening fatigue over 2 months. She had a 2-year history of Graves' disease (treated with iodine-131 1 year prior) and spotty facial pigmentation since the age of 10 years. Clinical examination revealed a left atrial myxoma, spotty pigmentation and Graves' disease-related hyperthyroidism. A diagnosis of CNC was established clinically based on the presence of two major criteria: a cardiac myxoma and characteristic lentigines. Preoperatively, the patient received 30 mg of methimazole daily and 10 mg of propranolol three times daily for 30 days to normalise her thyroid function. She subsequently underwent complete excision of the left atrial myxoma (dimensions: 6.5 × 4.5 × 2.5 cm) via a median sternotomy with a right atrial approach while under cardiopulmonary bypass. Postoperatively, antithyroid therapy was changed to 100 mg of propylthiouracil three times daily. No complications occurred after the operation.

**Conclusions:**

This is the first detailed report of a typical CNC case with concomitant, persistent Graves' disease, suggesting a potential novel association within the CNC spectrum. It underscores the importance of a multidisciplinary approach in managing the complex perioperative needs of patients with CNC.

## 1 Background

Carney complex (CNC), first described in 1985, is a rare autosomal-dominant multiple neoplasia syndrome with an incidence of approximately 1 in 200,000 individuals (1). Its core manifestations include spotty skin pigmentation (lentigines), cardiac myxomas, cutaneous myxomas and endocrine abnormalities (2). To date, over 500 cases have been registered in the National Institutes of Health, Mayo Clinic and Cochrane Centre databases (3).

Endocrine involvement is a key feature of CNC, with reported abnormalities including pituitary adenomas (acromegaly), primary pigmented nodular adrenocortical disease, testicular Sertoli cell tumours and thyroid lesions (4). Among these, thyroid abnormalities are relatively common, with over two-thirds of patients with CNC presenting with thyroid lesions. Most of these are non-hormone-secreting follicular adenomas, whereas thyroid cancer (e.g., papillary thyroid carcinoma) appears in approximately 5% of cases (5, 6). Graves' disease-related hyperthyroidism (an autoimmune-mediated hyperthyroid disorder) in combination with CNC has been rarely reported.

Cardiac myxomas are the most life-threatening manifestation of CNC and are responsible for 16% of specific mortality (resulting from complications such as tumour embolism, heart failure and arrhythmias) in families with a history of CNC (7). Unlike sporadic cardiac myxomas, which are usually solitary and occur in middle-aged women, CNC-related myxomas often occur in groups, are recurrent and may involve multiple cardiac chambers (8, 9). Surgical resection is the only curative treatment for cardiac myxomas, but the optimal surgical approach and perioperative management strategies for this procedure remain undefined, especially for patients with concurrent endocrine disorders such as hyperthyroidism (8).

The coexistence of Graves' disease and cardiac myxomas raises questions about potential associations: is their coexistence a coincidence, or does CNC have a pathogenic link to autoimmune thyroid disorders? Current hypotheses, which remain speculative, include shared genetic susceptibility [e.g., mutations in protein kinase cAMP-dependent type I regulatory subunit alpha (*PRKAR1A*), the major causative gene of CNC, may affect immune regulation] and tumour-induced autoimmune activation (e.g., myxoma-derived cytokines trigger thyroid autoimmunity (8, 9). However, limited clinical data hinder the validation of these hypotheses.

This case report aims to (1) describe a detailed case of typical CNC occurring in combination with Graves' disease with comprehensive clinical, radiological, pathological and follow-up data; (2) review the literature to explore plausible hypotheses for a potential association mechanism between CNC and Graves' disease; and (3) propose specific strategies for multisystem screening and long-term follow-up in patients with CNC, particularly those with thyroid abnormalities.

## 2 Case presentation

### 2.1 Demographic data and presenting features

The patient was an 18-year-old Chinese woman with a height of 162 cm, weight of 48 kg and body mass index of 18.3 kg/m^2^.

She presented with a 2-month history of heart palpitations and progressive fatigue, which had worsened over the preceding 2 weeks to the point of limiting daily activities such as stair climbing. The patient was diagnosed with Graves' disease at 16 years of age [confirmed by elevated free triiodothyronine (FT3) and free thyroxine (FT4) levels, suppressed thyroid-stimulating hormone (TSH) and a positive thyroid-stimulating hormone receptor antibody (TRAb) test]. She received oral methimazole (dose unknown) for 1 year, followed by iodine-131 therapy (8 mCi) 1 year prior to admission. After the iodine-131 therapy, the patient did not undergo regular thyroid function assessments.

The patient's parents were non-consanguineous. Her father died suddenly at the age of 30 from an unknown cardiac event (no autopsy performed). No other family members had a history of skin pigmentation, cardiac tumours or endocrine disorders. Genetic analysis (including *PRKAR1A* gene sequencing) was recommended to confirm CNC diagnosis, but the patient and her family refused due to concerns about the psychological impact of the results and financial constraints.

### 2.2 Physical examination (complete results)

**Figure 1:**
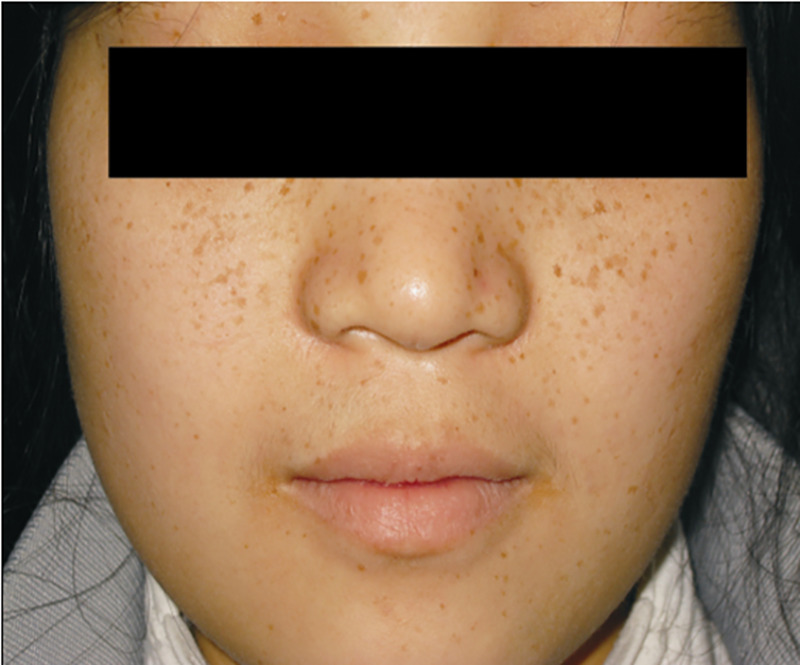
Spotty skin pigmentations were noted on her lips and perioral skin.

The results of the comprehensive physical examination are summarised in Table 1. Key positive findings included spotty mucocutaneous pigmentation (Figure 1), a diffusely enlarged thyroid gland and a systolic murmur at the mitral valve area. These findings provided strong clinical clues supporting the subsequent diagnoses of CNC and Graves' disease and were pivotal in guiding further imaging studies. The remainder of the systematic examination was unremarkable.

**Table 1:** Physical examination items and results.

Examination item	Findings	Reference range
Vital signs	Temperature: 36.5 °C; Pulse: 96 beats/min; Respiratory rate: 16 breaths/min; Blood pressure: 120/76 mmHg	Temperature: 36.0–37.2 °C; Pulse: 60–100 beats/min; Respiratory rate: 12–20 breaths/min; Blood pressure: 90–140/60–90 mmHg
Skin and mucosa	Lentigines (2–5 mm in diameter) on lips, infraorbital region, and conjunctiva (Figure 1); No xanthomas, petechiae, or skin myxomas; Skin turgor normal	No abnormal pigmentation or lesions
Head	Normocephalic; Hair distribution uniform, no alopecia	No abnormalities
Eyes	No proptosis (exophthalmometry: 12 mm bilaterally); No periorbital edema; Conjunctiva clear; Pupils equal and round (3 mm), light reflexes intact	Exophthalmometry: 10–14 mm; No proptosis
Ears, nose, throat	External ears normal; External auditory canals clear; Nasal mucosa intact; Pharynx non-erythematous; Tonsils not enlarged	No abnormalities
Neck	Thyroid gland diffusely and symmetrically enlarged (1.5× normal size), firm, non-tender, no nodules; No thyroid bruit; Cervical lymph nodes not palpable; Trachea midline	Thyroid non-enlarged; No bruit
Chest	Chest wall symmetric; Bilateral breath sounds clear, no rales or wheezes	No abnormalities
Heart	Apex beat at 5th intercostal space, left midclavicular line; Heart rate: 96 beats/min, regular rhythm; Grade 2/6 systolic murmur at mitral area (no radiation); No pericardial friction rub	No heart murmurs
Abdomen	Soft, non-tender; Liver and spleen not palpable; Bowel sounds: 4 times/min; No ascites (shifting dullness negative)	No abnormalities
Extremities	No clubbing, cyanosis, or edema; Radial and dorsalis pedis pulses symmetric	No abnormalities
Neurological	Muscle strength: 5/5 in all limbs; Deep tendon reflexes normal; No pathological reflexes	No abnormalities

### 2.3 Auxiliary examinations (ordered by diagnostic relevance)

#### 2.3.1 Laboratory tests

The laboratory test results are detailed in Table 2. Preoperative thyroid function assessments showed significantly elevated FT3 and FT4 levels, suppressed TSH and strong TRAb positivity, consistent with active Graves' disease. Postoperative monitoring showed a gradual normalisation of thyroid hormone levels.

**Table 2:** Laboratory test items and results.

Test item	Time point	Result	Reference range	Detection method
FT3	Preoperative Day 1	7.71 pmol/mL	3.1–6.8 pmol/mL	ECLIA
FT4	Preoperative Day 1	31.8 pmol/mL	12–22 pmol/mL	ECLIA
TSH	Preoperative Day 1	0.021 μIU/mL	0.27–4.2 μIU/mL	ECLIA
TRAb	Preoperative Day 1	18.6 IU/L	0–1.75 IU/L	ECLIA
TPOAb	Preoperative Day 1	32 IU/mL	0–34 IU/mL	ECLIA
TgAb	Preoperative Day 1	28 IU/mL	0–115 IU/mL	ECLIA
FT3	Postoperative Day 1	5.2 pmol/mL	3.1–6.8 pmol/mL	ECLIA
FT4	Postoperative Day 1	22.3 pmol/mL	12–22 pmol/mL	ECLIA
TSH	Postoperative Day 1	0.03 μIU/mL	0.27–4.2 μIU/mL	ECLIA
FT3	Postoperative Day 4	3.8 pmol/mL	3.1–6.8 pmol/mL	ECLIA
FT4	Postoperative Day 4	15.7 pmol/mL	12–22 pmol/mL	ECLIA
TSH	Postoperative Day 4	0.05 μIU/mL	0.27–4.2 μIU/mL	ECLIA
FT3	Postoperative Week 2	4.5 pmol/mL	3.1–6.8 pmol/mL	ECLIA
FT4	Postoperative Week 2	18.3 pmol/mL	12–22 pmol/mL	ECLIA
TSH	Postoperative Week 2	0.28 μIU/mL	0.27–4.2 μIU/mL	ECLIA
FT3	Postoperative Month 1	4.3 pmol/mL	3.1–6.8 pmol/mL	ECLIA
FT4	Postoperative Month 1	17.8 pmol/mL	12–22 pmol/mL	ECLIA
TSH	Postoperative Month 1	0.35 μIU/mL	0.27–4.2 μIU/mL	ECLIA
CBC	Preoperative Day 1	White blood cells: 5.6 × 10⁹/L; Hemoglobin: 135 g/L; Platelets: 220 × 10⁹/L	White blood cells: 3.5–9.5 × 10⁹/L; Hemoglobin: 115–150 g/L; Platelets: 100–300 × 10⁹/L	Automated hematology analyzer
Liver/kidney function	Preoperative Day 1	Alanine transaminase (ALT): 28 U/L; Aspartate transaminase (AST): 22 U/L; Creatinine: 65 μmol/L; Blood urea nitrogen (BUN): 4.2 mmol/L	ALT: 7–40 U/L; AST: 13–35 U/L; Creatinine: 41–81 μmol/L; BUN: 3.1–8.0 mmol/L	Automated biochemistry analyzer
Coagulation function	Preoperative Day 1	Prothrombin time (PT): 11.5 s; Activated partial thromboplastin time (APTT): 35.2 s; Fibrinogen: 2.8 g/L	PT: 10–14 s; APTT: 25–38 s; Fibrinogen: 2.0–4.0 g/L	Coagulation analyzer

FT3, free triiodothyronine; FT4, free thyroxine; TSH, thyroid-stimulating hormone; TRAb, thyroid-stimulating hormone receptor antibody; TPOAb, thyroid peroxidase antibody; TgAb, thyroglobulin antibody; ECLIA, electrochemiluminescence immunoassay; CBC, complete blood count; ALT, alanine transaminase; AST, aspartate transaminase; BUN, blood urea nitrogen; PT, prothrombin time; APTT, activated partial thromboplastin time.

#### 2.3.2 Imaging tests

A chest x-ray conducted on preoperative day 3 revealed a cardiothoracic ratio of 0.60 (≤0.50 is considered normal), mild cardiomegaly and no pulmonary infiltration.

**Figure 2:**
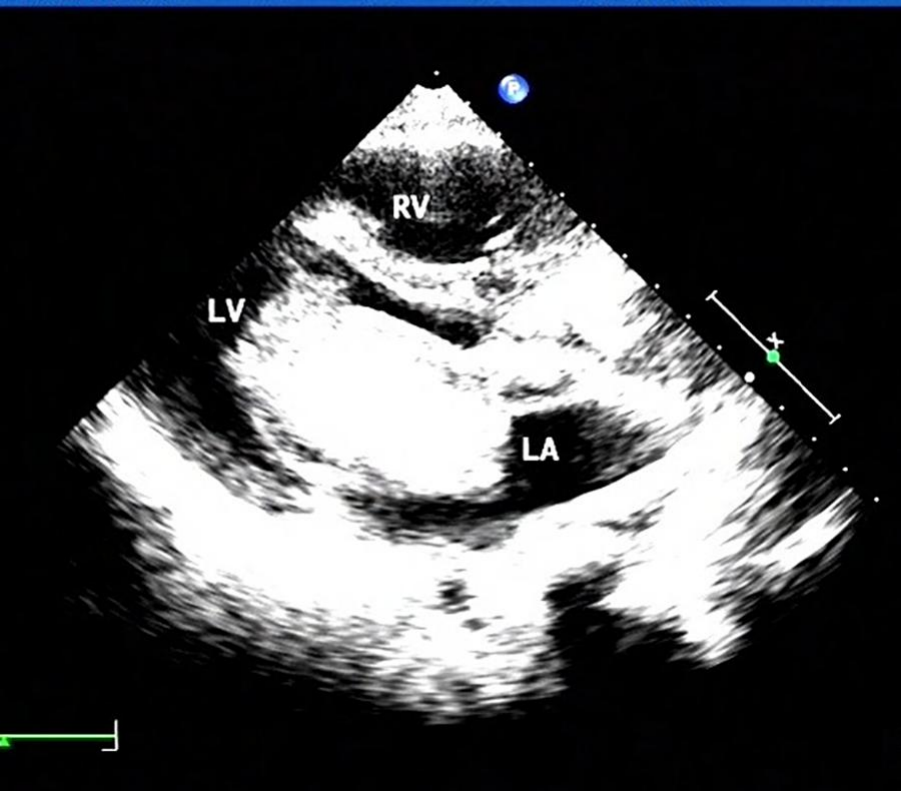
Transthoracic echocardiogram (apical four-chamber view) showing a large, hyperechoic atrial myxoma (arrow) attached to the interatrial septum.

A transthoracic echocardiogram (TTE) performed on preoperative day 2 revealed a normal sinus rhythm (heart rate: 96 beats/min), a hyperechoic mass (dimensions: 44 × 55 mm; attached to the interatrial septum near the fossa ovalis and almost obstructing mitral inflow), mild mitral regurgitation (Figure 2) and a left ventricular ejection fraction of 62% (≥50% is considered normal).

**Figure 3:**
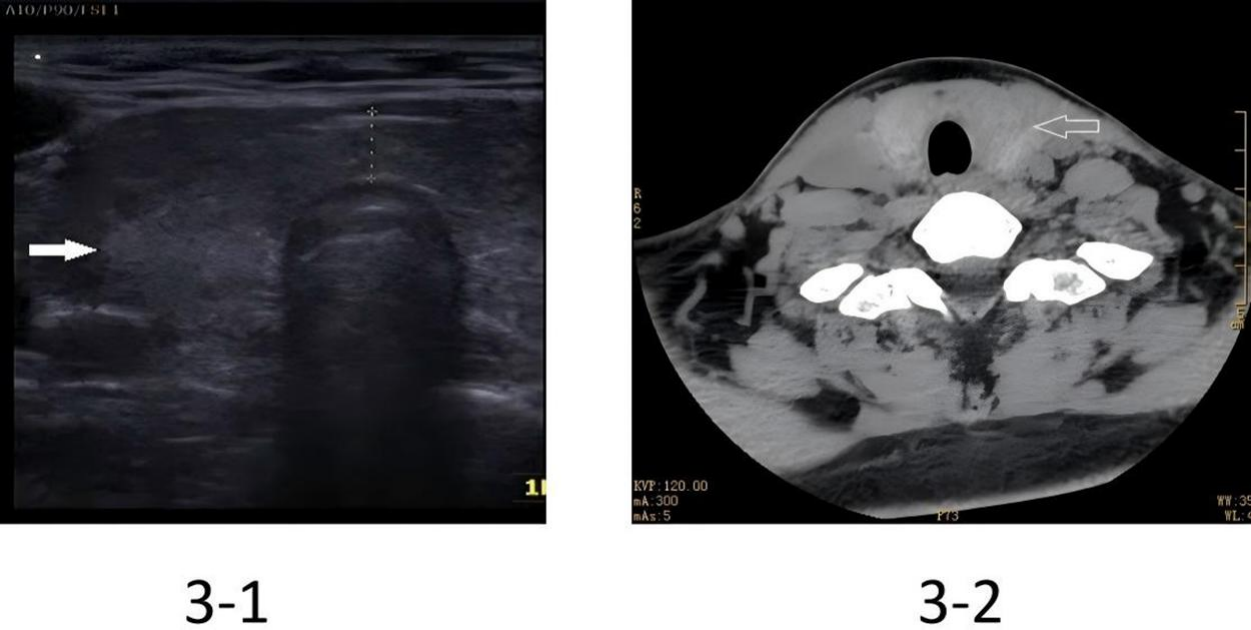
USG and CT scan revealed diffuse enlargement in the thyroid gland. Left: Thyroid ultrasound image; Right: Thyroid computed tomography image.

A thyroid ultrasound conducted on preoperative day 2 showed diffuse enlargement of the thyroid gland (right lobe: 5.2 × 2.1 × 1.8 cm; left lobe: 5.0 × 2.0 × 1.7 cm; normal reference: 4.0–5.0 × 1.5–2.0 × 1.0–1.5 cm), a homogeneous echo texture and no nodules or cystic lesions. A computed tomography scan of the thyroid performed on preoperative day 2 revealed diffuse thyroid enlargement with uniform enhancement and no tracheal compression (Figure 3).

#### 2.3.3 Pathological examination

**Figure 4:**
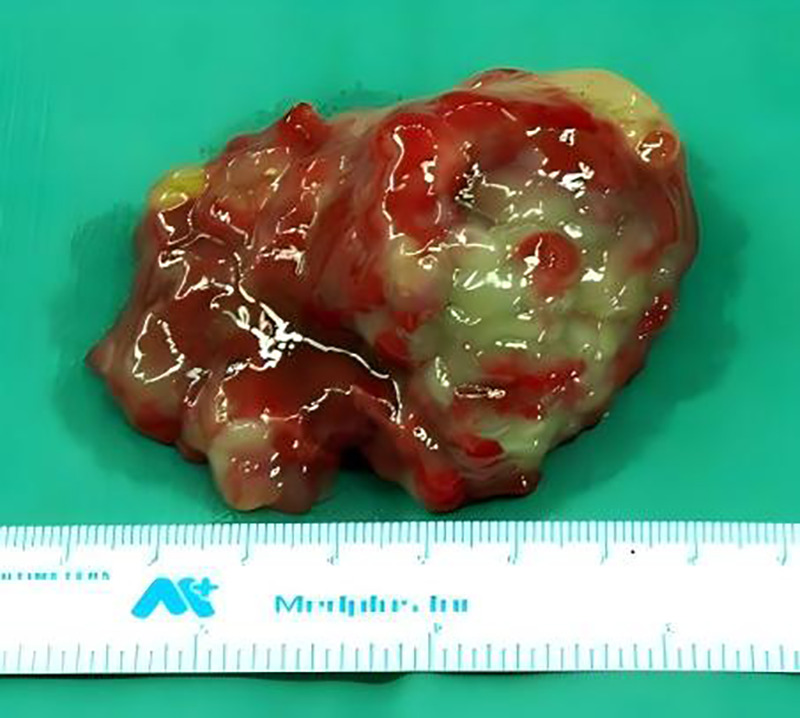
Intraoperative finding and pathological examination of the resected myxoma.

The gross features apparent upon examination included the following: the resected tumour was a solitary, smooth, egg-shaped mass (6.5 × 4.5 × 2.5 cm); the cut surface showed myxoid areas with congestion; and there was no apparent haemorrhage or necrosis (Figure 4).

### 2.4 Diagnostic process

#### 2.4.1 Diagnosis of Carney complex

The patient was diagnosed with CNC based on established clinical criteria (6), as she presented with two major manifestations of the condition: (a) a histologically confirmed cardiac myxoma and (b) the typical spotty skin pigmentation of CNC (lentigines). Although genetic confirmation was not obtained, the combination of these pathognomonic features was considered sufficient for a clinical diagnosis.

#### 2.4.2 Differential diagnoses

Sporadic cardiac myxoma was excluded owing to the patient's concurrent spotty pigmentation and her family history of sudden cardiac death (suggesting a genetic predisposition). Peutz–Jeghers syndrome was excluded because there were no gastrointestinal polyps, and no perioral pigmentation was evident. Likewise, McCune–Albright syndrome was excluded because there was no fibrous dysplasia of the bone and no history of precocious puberty.

Other causes of hyperthyroidism were excluded, as the positive TRAb test confirmed Graves' disease and excluded toxic multinodular goitre and thyroiditis as aetiologies of the patient's hyperthyroidism.

### 2.5 Treatment plan

#### 2.5.1 Preoperative preparation (30 days)

The patient's thyroid function was maintained with 30 mg of oral methimazole (once daily) to inhibit thyroid hormone synthesis and 10 mg of oral propranolol (three times daily) to control tachycardia. Thyroid function was monitored every 7 days until the FT3 and FT4 values returned to normal ranges. This was achieved on preoperative day 7.

For cardiac assessment, a TTE was conducted every 14 days to monitor the myxoma size and mitral inflow obstruction, with no notable changes noted.

#### 2.5.2 Surgical procedure: standardised protocol

A median sternotomy with a right atrial approach involving the incision of the right atrium and retraction of the interatrial septum was performed to expose and resect the left atrial myxoma while the patient was placed under cardiopulmonary bypass (CPB) for 78 min (aortic cross-clamp time: 42 min). The myxoma and its pedicle (attached to the interatrial septum) were completely resected, and the remaining interatrial septum defect was repaired with a bovine pericardial patch. Intraoperative monitoring was achieved using transoesophageal echocardiography, which confirmed complete resection of the tumour and normal mitral valve function, with no residual regurgitation.

#### 2.5.3 Postoperative management

Antithyroid therapy was switched from methimazole to propylthiouracil (100 mg three times daily) postoperatively. This change was instituted due to a pre-emptive concern about methimazole-associated hepatotoxicity in the perioperative setting, where drug-induced liver injury could complicate recovery. The dose was later tapered based on thyroid function assessments, registering at 50 mg three times daily by postoperative month 1.

2For cardiac care, postoperative ventilation was maintained for 5 h. The patient was transferred to the general ward on postoperative day 1, and low-molecular-weight heparin (4,000 IU once daily) was administered subcutaneously to prevent thrombosis.

### 2.6 Follow-up results

The patient was discharged on postoperative day 6. During the 4-year follow-up period, she exhibited no evidence of cardiac myxoma recurrence, maintained stable thyroid function on a tapering dose of antithyroid medication. Her skin pigmentation was unchanged, and no new cutaneous myxomas or other major CNC manifestations (pituitary or adrenal dysfunction et al.) were detected on systematic annual screening.

## 3 Discussion

### 3.1 Plausible hypotheses for an association between CNC and Graves' disease

The coexistence of CNC and persistent Graves' disease in this case suggests several non-mutually exclusive, hypothetical mechanisms. First, shared genetic susceptibility may play a role in this case. Carney complex is mainly caused by mutations in the *PRKAR1A* gene (10). This gene is located on chromosome 17q22-q24. Mutations in this gene lead to a dysregulation of the cAMP signalling pathway, a critical pathway for two processes: thyroid cell proliferation and the regulation of immune cell activation (11, 12). Abnormal cAMP activity could potentially enhance the production of thyroid autoantibodies; for example, it may increase TRAb levels by activating B lymphocytes (13). A 2018 study found that patients who carried a *PRKAR1A* mutation had a 3.2-fold higher risk of autoimmune thyroid disorders than members of the general population (14). Recent reviews on CNC pathogenesis suggest that PRKAR1A inactivation may have broader effects on immune homeostasis, though direct evidence linking it to Graves' disease is limited (15, 16).

Second, tumour-induced autoimmunity could also be a factor. Cardiac myxomas secrete certain cytokines, including interleukin-6 and tumour necrosis factor-α (17). These cytokines trigger systemic inflammation and also potentially promote autoimmunity (18, 19). A case report from 2023 describes a similar scenario, in which a patient with an atrial myxoma developed transient hyperthyroidism (20). The patient's hyperthyroidism was linked to cytokines from the myxoma and was resolved after tumour resection. In our case, the hyperthyroidism persisted postoperatively, suggesting that the myxoma may have exacerbated pre-existing Graves' disease rather than being the sole cause of the hyperthyroidism.

Third, although coincidence is a possible explanation in this case, the rarity of each condition (CNC incidence: approximately 1/200,000; Graves' disease prevalence in women: approximately 0.5%–1%) makes a purely coincidental association statistically improbable, although definitive epidemiological data to quantify this probability are lacking (14).

### 3.2 Mechanism of postoperative thyroid function fluctuations

The patient's thyroid function changed after surgery, first showing a transient decline, with FT3 and FT4 reaching their lowest levels on postoperative day 4, then gradually returning to normal. This pattern aligns with euthyroid sick syndrome (ESS), a common syndrome after cardiac surgery caused by several factors. One factor is the stress-induced inhibition of thyroid hormone conversion. Surgery and CPB trigger a stress response in which the body releases cortisol and catecholamines, substances that inhibit the conversion of T4 to T3 (T3 being the active form of the thyroid hormone). This conversion mainly occurs in the liver and kidneys, and its inhibition leads to a lowering of T3 levels. Another causal factor of ESS is reduced thyroid hormone binding. After surgery, acute-phase reactants, such as albumin, can change (e.g., albumin levels may decrease). Albumin binds to thyroid hormones in the blood; thus, lower albumin levels translate into less hormone binding. Although the levels of free hormones (e.g., FT3 and FT4) are less affected, this phenomenon still contributes to the occurrence of ESS (21).

Medication effects also play a role in ESS. The patient in the current report used propranolol perioperatively. Propranolol further inhibits the conversion of T4 to T3, adding to the transient reduction of FT3. A notable observation is the patient's suppressed TSH levels, which remained low until postoperative week 2. This may be due to persistent TRAb activity. Thyroid-stimulating hormone receptor antibody is an autoantibody that stimulates thyroid hormone secretion without relying on TSH. Thus, even in the context of low TSH, the thyroid still produces hormones.

### 3.3 Long-term follow-up recommendations for patients with Carney complex

Based on this case and recent guidelines, for patients with CNC, we propose specific follow-up strategies covering multiple systems. For the cardiac system, TTEs are essential. In the first 5 years, patients should undergo a TTE every 6 months. After 5 years, if no recurrence occurs, an annual TTE is sufficient (22). For the thyroid system, two types of monitoring are needed: thyroid function assessments (including FT3, FT4, TSH and TRAb), which should be performed every 3 months in the first year and every 6 months thereafter, and thyroid ultrasound, which should be performed annually to screen for thyroid nodules and cancer. For the integumentary system, regular clinical examinations are necessary. Doctors should check for lentigines or myxomas every 6 months. Genetic testing is also recommended; *PRKAR1A* gene testing should be offered to patients and their first-degree relatives to help identify asymptomatic carriers. Early identification can lead to timely intervention.

### 3.4 Study limitations

This study has several limitations that need to be acknowledged. First, there is a lack of genetic testing. The patient refused *PRKAR1A* gene sequencing; thus, we cannot verify a genetic basis for her CNC. This limits our ability to explore genotype–phenotype correlations. For example, we cannot check whether specific *PRKAR1A* mutations increased her risk of Graves' disease.

Second, this study uses a single-case design. Case reports cannot establish causality; we cannot confirm that CNC causes Graves' disease. Multicentre cohort studies are needed to validate an association between the two diseases.

Third, the long-term follow-up period of this study is limited, and a longer follow-up period (preferably 10 years or more) is needed to assess the risk of myxoma recurrence and thyroid cancer more accurately.

Finally, due to the retrospective nature of this study design, the pathological diagnosis lacked the immunohistochemical characteristics of myxoma.

Future research directions are clear: *PRKAR1A* mutation screening in more patients with coexisting CNC and Graves' disease is needed, and long-term multicentre cohort studies are required to help clarify the association between these two diseases and provide more evidence for clinical management strategies.

## 4 Conclusion

This report describes the first detailed case of typical Carney complex with concomitant, persistent Graves' disease, suggesting that Graves' disease may represent a potential novel association within the CNC spectrum. This finding underscores the importance of comprehensive endocrine and cardiac evaluation in patients with either condition. Whether this association is causal or reflects shared pathogenesis remains to be elucidated by future research, but it carries significant implications for the multidisciplinary management and long-term prognosis of such complex patients.

## Data Availability

The original contributions presented in the study are included in the article/Supplementary Material, further inquiries can be directed to the corresponding author/s.
